# Association of *H-FABP* gene polymorphisms with intramuscular fat content in Three-yellow chickens and Hetian-black chickens

**DOI:** 10.1186/s40104-016-0067-y

**Published:** 2016-02-18

**Authors:** Yong Wang, Xiaohong Hui, Huie Wang, Tursunjan Kurban, Chao Hang, Ying Chen, Jinming Xing, Jiufeng Wang

**Affiliations:** College of Veterinary Medicine, China Agricultural University, Beijing, 100193 China; Key Laboratory of Tarim Animal Husbandry Science & Technology of Xinjiang Production and Construction Groups/College of Animal Science, Tarim University, Alar, Xinjiang Uygur Autonomous Region 843300 China

**Keywords:** Hetian-black Chicken, H-FABP, mRNA Expression, Polymorphism, Three-yellow Chicken

## Abstract

**Background:**

To explore the relationship between the heart-type fatty acid binding protein (H-FABP) gene and intramuscular fat (IMF), a polymorphism of the second exon of the *H-FABP* gene was investigated in 60 Three-yellow chickens (TYCs) and 60 Hetian-black chickens (HTBCs).

**Results:**

The IMF contents of the cardiac, chest and leg muscles in HTBC were increased compared with TYC. Both TYC and HTBC populations exhibited Hardy-Weinberg Equilibrium (HWE) according to the χ^2^ test. Three variations of the two birds were detected, namely, G939A, G982A and C1014T. HTBCs with the TT genotypes exhibit increased IMF content in the chest muscles compared with the TC genotype. Thus, the G982A site could be considered a genetic marker for selecting increased IMF content in the chest muscles of HTBC. The correlation coefficients revealed that *H-FABP* mRNA expression was negatively correlated with the IMF content in the cardiac, chest and leg muscles of HTBC and in the cardiac and chest muscles of TYC. The relative mRNA expression of *H-FABP* was reduced in the cardiac and leg muscles of HTBC compared with TYC, but this difference was not observed at the protein level, as assessed by Western blot analysis.

**Conclusions:**

These findings offer essential data that can be useful in the breeding program of HTBC and future research exploring the role of H-FABP in IMF deposition and regulation in chickens.

## Background

Meat quality is one of the most important factors influencing the acceptability of meat [1]. However, the quality and flavor of chicken have decreased in the past decades as a result of genetic selection for faster growth velocity and increased feed conversion efficiency [2]. This phenomenon is particularly evident in China and many Southeast Asian countries and regions [3]. However, people prefer to consume the traditional slow-growing, meat-type, colored-feather chickens in many regions of the world [4]. These traditional chickens, which mainly include local varieties, are also popular in China, and the market share of these as meat birds is as high as 50 % [5].

The Hetian-black chicken (HTBC) is a type of slow-growing chicken with excellent meat taste and black feathers. Due to its rare genetic resources, it was included in the Directory of National Animal Genetic Resources in 2010. The HTBC has a history spanning more than 1750 years and is only distributed in the townships of Minfeng County of the Xinjiang Uygur Autonomous Region in China. Through attempts to improve the slow growth and low feed conversion rate, the HTBC was greatly hybridized with other chickens, and the pure breed is now in danger of extinction. In 2007, only 5,700 birds remained according to the Animal Genetic Resources of China. Three-yellow chicken (TYC), a fast-growing chicken, is widely farmed in China as a major meat-type broiler.

Intramuscular fat (IMF), which is the fat or lipid content extracted from muscle [5], has become one of the most important indicators of the quality of meat [2, 3, 6, 7]. Previous studies demonstrated that IMF influences the quality traits of meat [1, 2]. In addition, IMF is associated with the juiciness of beef and the flavor of pork [8, 9]. Moreover, moderate heritability and genetic selection for IMF have been utilized to improve meat quality in selection programs for swine [10, 11]. However, few reports have assessed the relationship between IMF deposition and the genes related to fat deposition in HTBC.

IMF content in muscle is related to the expression of lipogenic generation [1]. For example, the fatty acid binding protein (FABP) gene belongs to a supergene family of hydrophobic ligand-binding proteins [12, 13] and is composed of low-molecular-mass proteins that bind fatty acids [14]. FABPs have been isolated from several tissues of invertebrates and vertebrates [15, 16]. Nine types of FABPs have been identified in the mammalian FABP family [17–20]. Interestingly, the same types of FABP can be noted in more than one organ, and most tissues express various types of FABPs [21]. The FABPs have similar molecular weight [13] and similar molecular structure [18]. These proteins participate in transporting water insoluble fatty acids from the plasmalemma to the location of β-oxidation in the mitochondria as well as transporting other hydrophobic ligands [16, 21, 22]. Moreover, FABPs protect enzymes from the detergent-like effects of free fatty acids, modulate enzyme activity and gene transcription, and have signal transduction functions [12, 13, 15]. However, the precise functions of FABPs have not yet been fully elucidated [16, 22].

Of these genes, *H-FABP* was studied as a candidate gene to determine the IMF content for the evaluation of meat quality [2, 12, 15, 16, 20, 23]. Polymorphisms in the first exon and the first intron of the *H-FABP* gene in chickens are reportedly correlated with the IMF content [24]. A previous study demonstrated that *H-FABP* was the central gene involved in the fatty acid and fat metabolism of chickens [25]. *H-FABP* is related to the absorption of fatty acids and the promotion of effective fat storage and utilization [24, 26, 27]. *H-FABP* is also important in the development and adipogenic differentiation of stromal-vascular cells [1].

In addition, the relationship between *H-FABP* polymorphisms and expression and IMF has not been demonstrated in TYCs and HTBCs. Therefore, the aim of the current study was to explore the association of IMF and *H-FABP* gene polymorphisms and expression levels in these two chicken breeds. These findings will offer essential molecular information that can be used to explore the role of *H-FABP* in IMF deposition and regulation in chickens.

## Materials and methods

### Animals

The protocol for the animals in the current study was approved by the Tarim University Institutional Animal Care and Use Committee (TARU -ACUC-2012-051). All of the breeding HTBC specimens were collected from the breed’s sole provenance, Minfeng County. All of the TYC and HTBC specimens used in our experiment were maintained under the same environmental conditions at the Tarim University experimental station for animals, including *ad libitum* access to food and water. The commercial diets used in the current study met all National Research Council (NRC) requirements [28]. All treatments for the animals were in accordance with the Institute for Laboratory Animal Research (ILAR) Guide for the Care and Use of Laboratory Animals. A total of 120 birds divided into two groups were hatched and reared from 1 d until their slaughtering ages (70 d for TYC and 120 d for HTBC). 60 TYC specimens and 60 HTBC specimens with a 1:1 sex ratio were selected randomly and then anesthetized and sacrificed by exsanguination.

### IMF content

The IMF contents in the cardiac, chest and leg muscles were measured using the Soxhlet petroleum-ether extraction method according to Chinese National Standards GB/T 5009.6.2004, and the IMF content was determined as a weight percentage.

### Polymerase chain reaction-single-strand conformation polymorphism (PCR-SSCP)

Blood samples taken from the wing vein were anticoagulated with acid citrate dextrose (ACD) and stored at −20 °C for DNA extraction. The PCR was performed using a typical 20 μL system containing 10 μL 2 × SG PCR MasterMix (Beijing SinoGene Scientific Co. Ltd., China), 1 μL DNA, 8 μL dd H_2_O, and 0.5 μL primers (10 μmol/L) (F: 5’-CGACAAGGCGACGGTGAA-3’; R: 5’-TGGGGCAGGAAGGAGTTT-3’) (accession number: AY648562). The amplification conditions were as follows: pre-denaturation at 94 °C for 3 min; 35 cycles of denaturation at 94 °C for 30 s, annealing at 60 °C for 30 s and extension at 72 °C for 30 s; and a final extension at 72 °C for 10 min. The PCR products were detected on 1 % agarose gel. A 50 μL expansion system was used to recover the products.

The polymorphism of the *H-FABP* gene second exon was detected via PCR-SSCP. The PCR products were combined with PCR–SSCP buffer containing 0.1 % bromophenol blue and 0.1 % xylene cyanol in formamide. Then, the mixtures were degenerated for 10 min at 98 °C and maintained on ice for 5 min. Each sample was transferred to a 12 % polyacrylamide gel with 10 × TBE buffer. The gels were run at 4 °C under the following conditions: 250 V for 10 min and 56 V for 16 h. The gels were stained according to a standard protocol [24]. Homozygotic type fragments were cut under an ultraviolet lamp and then purified with a DNA purification kit. The recovered DNA fragments were linked with pMD18-T Simple Vector and then transformed into a DH5α strain. The positive clones were selected and identified by PCR and then sequenced using TaKaRa (TaKaRa Biotechnology Inc., Dalian, China).

### Quantitative real-time PCR (qPCR)

Total RNA was extracted from the cardiac, chest and leg muscle tissue samples using TRIzol reagent (Invitrogen, Carlsbad, CA), as previously described [29]. The integrity of the RNA extracted from each sample was confirmed by agarose gel electrophoresis with ethidium bromide staining and visualization under ultraviolet (UV) light. A NanoDrop® ND-2000C spectrophotometer (Thermo Fisher Scientific, Wilmington, DE) was used to determine the amount of RNA extracted and verify its purity (OD_260_/OD_280_ absorption ratio > 1.9). Next, 1 μg of total RNA was reverse transcribed into first-strand cDNA using the GoScript reverse transcription system (Promega, Madison, WI). To control for DNA contamination, a negative control (without enzyme) was included. The synthesized cDNA was stored at –20 °C prior to real-time PCR analysis.

An ABI 7500 Real-time PCR System (Applied Biosystems, Foster City, CA) was used for qPCR analyses. The sequences of the primers used are listed below: *H-FABP*, F: 5’-CAGAAGTGGGATGGGAAGGAGA-3’, R: 5’-TCATAGGTGCGGGTGGAGAC-3’ (accession number: NM204290); *β-actin* (housekeeping gene), F: 5’-AACACCCACACCCCTGTGAT-3’, R: 5’-TGAGTCAAGCGCCAAAAGAA-3’ (accession number: L08165). The cDNA was amplified with SYBR® Premix DimerEraser^TM^ (TaKaRa Biotechnology Inc., Dalian, China) containing 2 μL of cDNA, 1.0 μmol/L primers, 10 μL of 2 × SYBR Premix DimerEraser and 0.4 μL of ROX (passive reference dye). A non-template control of nuclease-free water was included in each run. All reactions were conducted in triplicate. The reaction was performed as follows: 1 cycle of 95 °C for 30 s; 39 cycles of 95 °C for 5 s, 60 °C for 30 s, and 72 °C for 60 s; and 1 cycle of 95 °C for 15 s, 60 °C for 60 s, 95 °C for 30 s, and 60 °C for 15 s. To quantify the relative mRNA expression, the cycle threshold (C_T_) values of the target genes were normalized to the C_T_ value of the housekeeping gene, and the results are presented as the fold change using the 2^−ΔΔCT^ method. The relative expression of the target gene mRNA in each group was calculated using the following equations: ΔC_T_ = C_T target gene_ ‐ C_T housekeeping gene_ and ΔΔC_T_ = ΔC_Ttreated group_ ‐ ΔC_Tcontrol group_.

### Western blotting

Frozen tissue samples (0.1 g) were ground with protease inhibitors (1 μg/mL leupeptin, 1 μg/mL pepstatin A, and 2 μg/mL aprotinin) using a glass grinder on ice. The lysates were centrifuged to remove the insoluble material. The supernatant was collected, and the protein concentration was measured using a protein assay (Bio-Rad Laboratories, Hercules, CA, USA). Sodium dodecyl sulfate-polyacrylamide gel electrophoresis (SDS-PAGE) sample buffer (10 mmol/L Tris-HCl, pH 6.8, 2 % SDS, 10 % glycerol, 0.2 mol/L dithiothreitol (DTT)) was added to the lysates. The mixture was heated at 100 °C for 5 min, followed by centrifugation at 15,000 × g for 15 min at 4 °C to remove the insoluble debris. The supernatant was used for Western blot analysis. A total of 50 μg of protein was loaded into each well in a 10 % SDS-PAGE gel. The resolved proteins were transferred onto nitrocellulose and blocked with 5 % non-fat milk. An anti-cardiac FABP primary antibody (Abcam, Cambridge, UK) was used at a dilution of 1:500 at 4 °C overnight. The blots were thoroughly washed and then exposed to goat anti-rabbit IgG HRP (M21002; Abmart) at a dilution of 1:1,000 for 1 h at room temperature. Finally, the signal was detected using an enhanced chemiluminescence (ECL) kit. β-actin (4970; Cell Signal) in each sample was amplified as a housekeeping control as presented in the lower panel.

### Statistical analysis

The frequencies of alleles and genotypes were analyzed using the POPGENE software package (v.1.31), and the PowerMaker software package (v.3.25) was used to analyze the polymorphic information content (PIC).

The correlation between the *H-FABP* genotypes and IMF content was performed using the SAS statistical software package, version 9.0 (SAS Institute, Inc., Cary, NC, USA) using the SAS software PROC GLM procedures. The following statistical model was applied: Y = μ + G + S + f + h + e, where Y = the dependent variable, μ = the population mean, G = fixed effects of the breed, S = fixed effects of sex, f = family, h = random effects, and e = random error. The G × S interaction was not significant for any trait and therefore was not included in the model.

Statistical analyses of the mRNA differential expression were conducted with the SPSS statistical software package, version 17.0 (SPSS Inc., Chicago, IL, USA) using the independent samples *t*-test. The correlation between the mRNA expression (2^−ΔCt^) [10] and the IMF content was assessed by Pearson’s correlation coefficient [30]. The difference was considered significant at *P* < 0.05 unless otherwise specified.

Statistical analysis of the H-FABP protein expression in different chickens was performed with Quantity One (v. 4.62). SPSS (17.0) was utilized to analyze the significant difference between two birds through the independent-samples *t*-test.

## Results

### IMF content

**Fig. 1:**
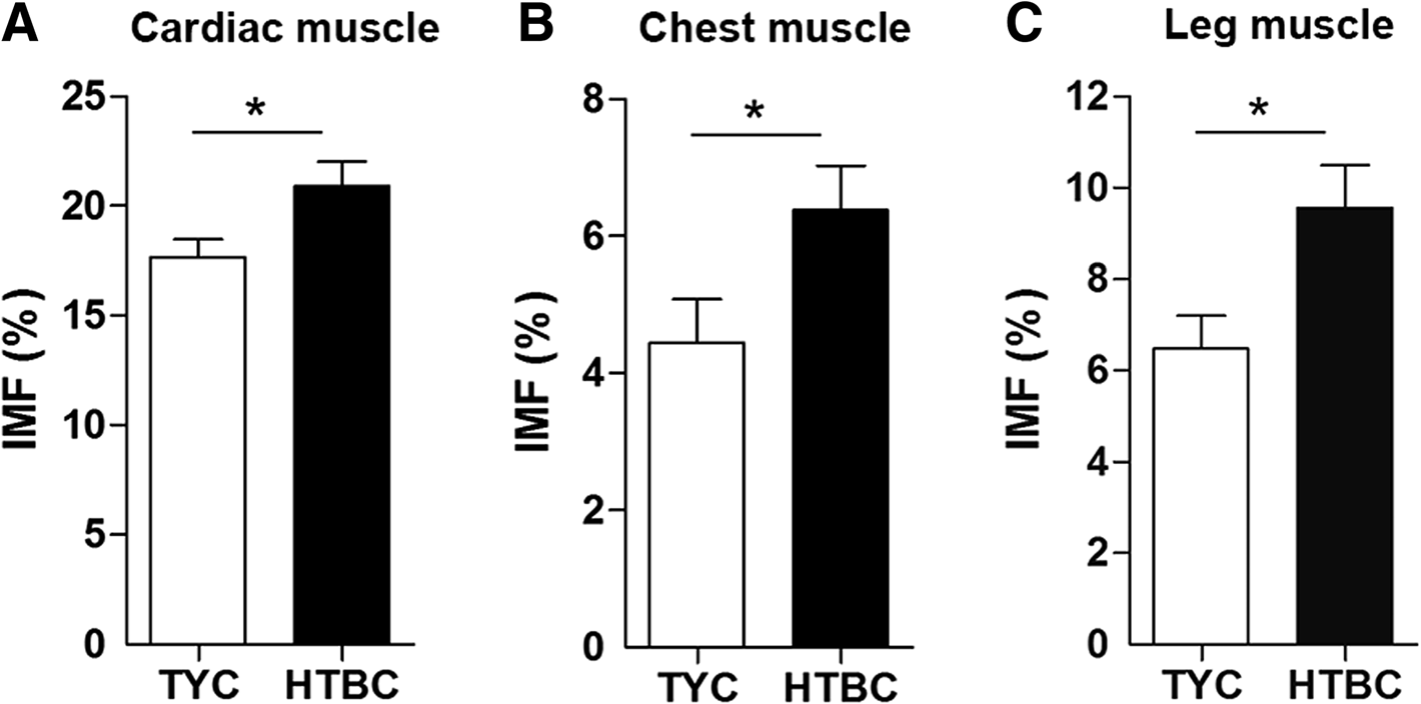
IMF content in cardiac muscle, chest and leg muscles of TYC and HTBC. Cardiac, chest and leg muscles were collected from TYC at 70 d and HTBC at 120 d. The IMF contents of cardiac (**a**), chest (**b**) and leg (**c**) muscles were measured via the Soxhlet petroleum-ether extraction method according to Chinese National Standards GB/T 5009.6.2004. The data are presented as the mean ± standard error of the mean (SEM) for each tissue (*n* = 60 per group). ^*^ *P* < 0.05. IMF, intramuscular fat; TYC, Three-yellow chicken; HTBC, Hetian-black chicken

Contents of IMF in two chicken populations are shown in Fig. 1. IMF contents in the cardiac, chest and leg muscles of HTBC were increased compared with those in TYC (*P* = 0.028, *P* = 0.047, *P* = 0.016, respectively).

### H-FABP gene polymorphism

**Fig. 2:**
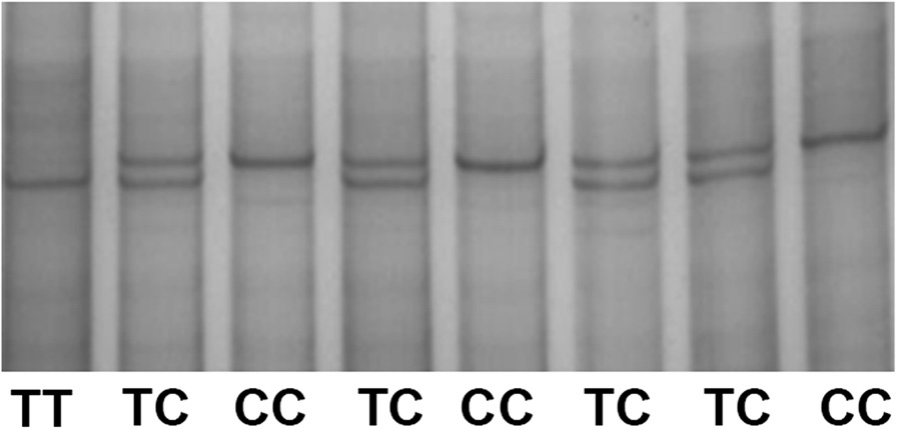
*H-FABP * genotypes of TYC and HTBC. Blood samples were collected from the wing vein of TYC at 70 d and HTBC at 120 d, anticoagulated via acid citrate dextrose (ACD), and DNA was extracted. Polymerase chain reaction-single strand conformation polymorphism (PCR-SSCP) was performed to analyze the polymorphism of the second exon of the *H-FABP* gene. The bands were named as TT, TC and CC

The results of PCR-SSCP indicated that the *H-FABP* gene had three types of single-strand conformation polymorphism (SSCP) bands: TT, TC and CC (Fig. 2). Genotypic frequency and gene frequency analyses revealed that TT was the dominant genotype and that T was the dominant allele of both TYC and HTBC in natural selection (Table 1). The sequences of TT and CC genotypes were compared with the reference sequence (AY648562) registered in GenBank. Three identical mutation sites were identified in *H-FABP* exon 2 in the two birds: G939A, G982A and C1014T.

**Table 1:** Genotypic and gene frequency of *H-FABP* in TYC and HTBC

Breed	Genotypic Frequency, %			Gene Frequency, %	
	TT	TC	CC	T	C
TYC	0.467 (28/60)	0.400 (24/60)	0.133 (8/60)	0.667 (80/120)	0.333 (40/120)
HTBC	0.417 (25/60)	0.383 (23/60)	0.200 (12/60)	0.608 (73/120)	0.391 (47/120)

*H-FABP* heart-type fatty acid binding protein, *TYC* Three-yellow chicken, *HTBC* Hetian-black chicken

TT, TC and CC are genotype frequencies; T and C are alleles. These genotypes were analyzed using the POPGENE software package (v.1.31) in 60 chickens with a 1:1 sex ratio in each group

The genetic polymorphism parameters are presented in Table 2. Both values of expected heterozygosity (He) were higher than those of the observed heterozygosity (Ho), and both of the Polymorphic information contents (PICs) of the two breeds were in the range 0.25 < PIC < 0.5.

**Table 2:** Hereditary character of *H-FABP* in TYC and HTBC

Breed	Ho	He	Ne	PIC	χ^2^	*P*
TYC	0.400	0.448	1.800	0.346	0.708	0.40
HTBC	0.383	0.481	1.910	0.364	2.500	0.11

*H-FABP* heart-type fatty acid binding protein, *TYC* Three-yellow chicken, *HTBC* Hetian-black chicken

*Ho* observed heterozygosity, *He* expected heterozygosity, *Ne* effective number of alleles, *PIC* polymorphic information content

Ho, He, Ne and χ^2^ were analyzed using the POPGENE software package (v. 1.31), and the PIC was analyzed using the PowerMaker software package (v. 3.25)

The χ^2^ values were 0.708 (*P* = 0.400) in TYC and 2.500 (*P* = 0.114) in HTBC. The populations of both chicken breeds exhibited Hardy-Weinberg equilibrium (HWE).

### Association between the *H-FABP* gene polymorphism and IMF content

The results of the association analysis between genotypic frequency and IMF content are displayed in Table 3. HTBC specimens with the TT genotype exhibited increased IMF content in the chest muscles compared with the TC genotype (*P* = 0.035) based on the least-square mean.

**Table 3:** Relationship between *H-FABP* polymorphism and IMF content in TYC and HTBC

Tissue	Breed	TT	TC	CC
Chest muscle	TYC	5.029 ± 0.307	3.994 ± 0.332	4.314 ± 0.575
	HTBC	7.535 ± 0.568^a^	5.457 ± 0.581^b^	6.133 ± 0.804^ab^
Leg muscle	TYC	7.301 ± 0.673	5.628 ± 0.727	6.506 ± 1.259
	HTBC	10.643 ± 1.031	8.742 ± 1.053	9.320 ± 1.458

*H-FABP* heart-type fatty acid binding protein, *TYC* Three-yellow chicken, *HTBC* Hetian-black chicken

The correlation between *H-FABP* gene polymorphism and IMF content was performed using the SAS 9.0 software package’s PROC GLM procedures

^a,b^Means within a row with no common superscript are different (*P* < 0.05)

### Correlation between *H-FABP* gene mRNA expression and IMF content

**Fig. 3:**
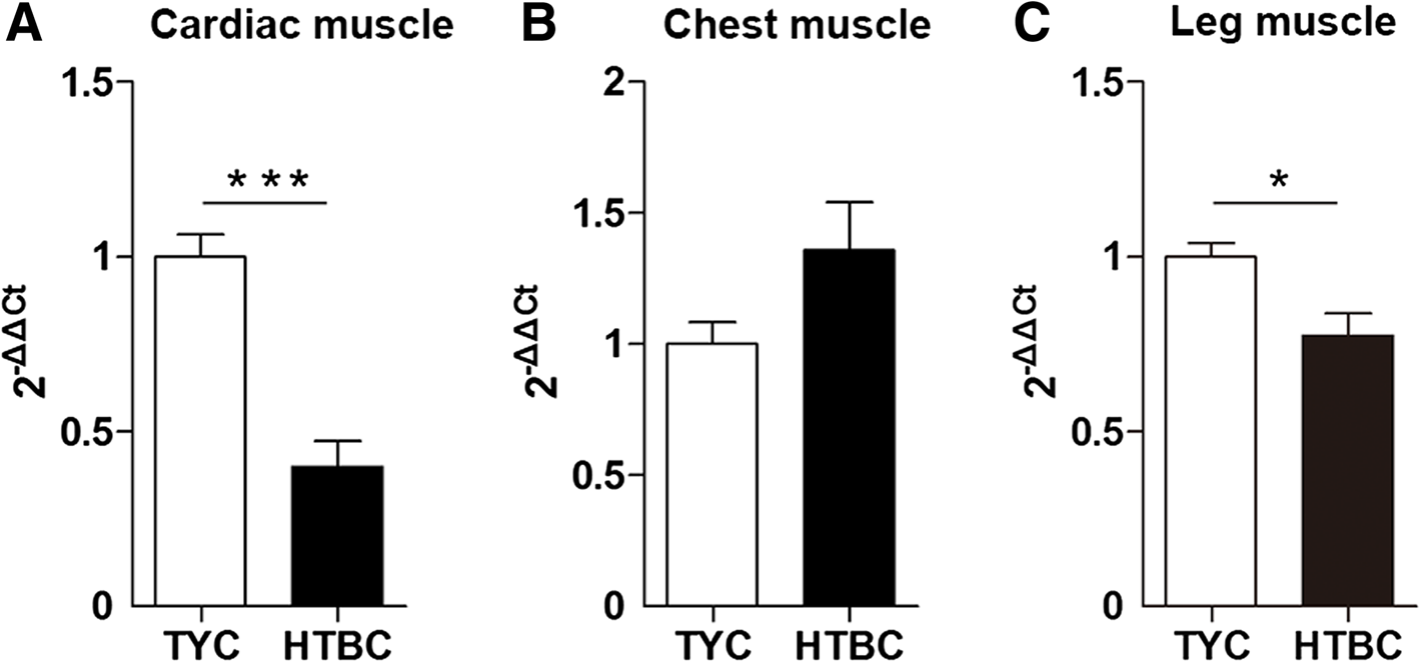
Relative expression of *H-FABP* mRNA in cardiac, chest and leg muscles of TYC and HTBC. Cardiac, chest and leg muscles were collected from TYC at 70 d and HTBC at 120 d. The relative expression of mRNA for the gene encoding *H-FABP* in cardiac (**a**), chest (**b**) and leg (**c**) muscles was analyzed using quantitative real-time PCR. The data are presented as the mean ± SEM for each tissue (*n* = 60 per group). ^*^ *P* < 0.05; ^***^ *P* < 0.001. TYC, Three-yellow chicken; HTBC, Hetian-black chicken

The relative *H-FABP* gene expression in different tissues in these two chicken breeds is presented in Fig. 3. In the cardiac and leg muscles, the expression of *H-FABP* mRNA in HTBC was reduced compared with that in TYC (*P* < 0.001 and *P* = 0.02, respectively). No difference was noted in chest muscle *H-FABP* mRNA expression in the two chicken breeds.

The association coefficients of the *H-FABP* gene mRNA with the IMF contents in the cardiac, chest and leg muscles were -0.588 (*P* = 0.045), -0.649 (*P* = 0.012) and -0.441 (*P* > 0.05) in TYC and -0.667 (*P* = 0.018), -0.646 (*P* = 0.023) and -0.608 (*P* = 0.030) in HBTC, respectively. Negative correlations were observed in these tissues with the exception of the leg muscle in TYC (Table 4).

**Table 4:** Correlation between *H-FABP* mRNA expression and IMF content in TYC and HTBC

Tissue	TYC	HTBC
Cardiac muscle	−0.588^*^	−0.667^*^
Chest muscle	−0.649^*^	−0.646^*^
Leg muscle	−0.441	−0.608^*^

*H-FABP* heart-type fatty acid binding protein, *TYC* Three-yellow chicken, *HTBC* Hetian-black chicken

The correlation analysis between mRNA expression (2^−ΔCt^) and IMF content was assessed by Pearson’s correlation coefficient at slaughter time using the SPSS 17.0 software package, ^*^ *P* < 0.05

**Fig. 4:**
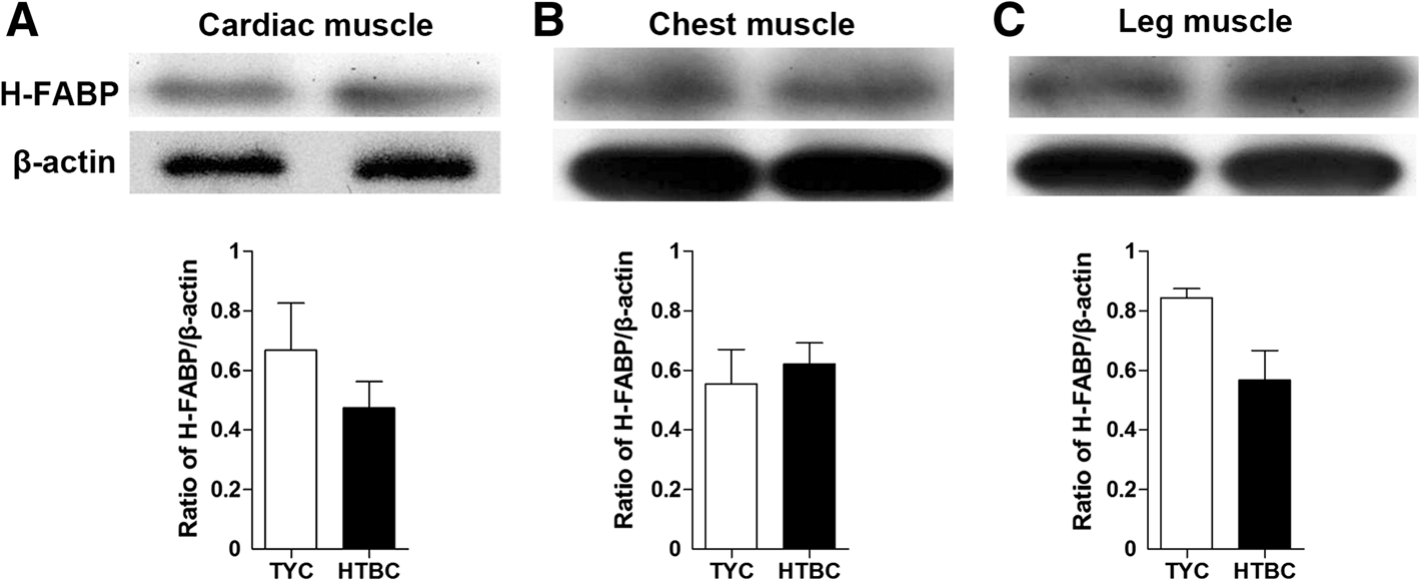
H-FABP protein expression in cardiac, chest and leg muscle in TYC and HTBC. Cardiac, chest and leg muscles were collected from TYC at 70 d and HTBC at 120 d. H-FABP protein expression in cardiac (**a**), chest (**b**) and leg (**c**) muscles was detected by western blot. Representative panels of H-FABP protein are shown. Expression of β-actin was measured as an internal control. The intensities of H-FABP and β-actin bands were determined using the Quantity One software package. The results are presented as the ratio of the H-FABP band intensity to the β-actin band intensity. The data are presented as the mean ± SEM for each tissue (*n* = 4 per group). H-FABP, heart-type fatty acid binding protein; TYC, Three-yellow chicken; HTBC, Hetian-black chicken

Interestingly, the trend of H-FABP protein expression in the different muscles mirrored the changes in H-FABP mRNA expression, but no statistical significance was observed at the protein level (Fig. 4).

## Discussion

Considerable efforts have been made to improve the speed of growth, daily weight gain and feed efficiency in chicken breeding over a long period of time. However, increased productivity led to dramatically decreased meat quality [2, 24]. As people’s quality of life has improved, higher requirements have been placed on the meat quality of chickens. Improving meat quality has become an important aim of breeding to meet people’s increased living standard. With their excellent meat and unique flavor, HTBCs have received considerable attention, but the native Chinese breed is going to become extinct. Therefore, the mechanism underlying the wonderful HTBC meat quality should be investigated immediately.

The growth and development process of fatty tissue is very complex because the procedure is associated with a variety of genes and pathways [31]. The IMF content, which refers to the deposition of fat within the muscles, affects the toughness of pork by changing the structure of the connective tissue [8] and affects the flavor and juiciness of chicken meat [32]. In the three tissues mentioned in the present experiment, the IMF contents in HTBC were increased compared with those in TYC, which could be the reason that slower-growing chickens have better flavor and meat quality than faster-growing chickens. The fact that HTBC meat is more popular than TYC meat in markets is consistent with this result. These results are consistent with the findings of Tu *et al.*, who reported a similar phenomenon in Rugao and Luyuan chickens [10].

Fatty acid binding proteins expressed in mammalian tissues or cells serve as intracellular transporters to satisfy special cellular needs [13, 18]. Members of the FABP families are thought to be closely related to IMF deposition. Of these, H-FABP is detected in many species, ranging from arthropods to mammals. In addition, H-FABP plays a critical role in determining the IMF content [16]. In chickens, the *H-FABP* gene located on chicken chromosome 23 is composed of 3 introns and 4 exons that code for 132 amino acids. This gene is expressed in various types of tissues, such as liver, muscle and heart [14]. H-FABP is essential for the binding of long-chain fatty acids and the transportation of fatty acids from the cell membrane to the sites of fatty acid oxidation and triglyceride and phospholipid synthesis [33–35].

The results of the χ^2^ test indicated that the two populations in this study were in HWE, which could be a consequence of long-time natural and artificial selection [36]. Both He values were higher than the Ho values, indicating more homozygous samples than heterozygote samples. The PICs of both breeds were in the range of 0.25 < PIC < 0.5, thereby indicating that moderate polymorphisms were detected at this locus.

The autogenous variation of *H-FABP* has an important influence on IMF deposition and other biological traits of chickens [24]. The assay of PCR-SSCP had been used to analyze polymorphisms for cows [37], pigs [38] and chickens [39]. Ye *et al.* [40] assessed a SNP (C2054T) in the second intron of the Beijing-Oil chicken *H-FABP* gene that remarkably correlated with the IMF contents in the chest and leg muscles. Eight SNPs (G332A, G534A, C835T, -1131A, C1294A, C2329T, C2372T, and C2636T) in the *H-FABP* gene of Caoke chickens were detected and correlated with carcass traits. Their results indicated that the genotypes of one primer pair exhibited a significant difference in the half-eviscerated weight, body weight, chest weight, thigh weight and carcass weight; thus, *H-FABP* could have a strong impact on carcass traits or could be connected with genes that affect slaughter performance in chickens. Four SNPs (C260T, A675G, C783T, and A2778G) in the *H-FABP* gene in Fengkai Xinghua, Huiyang Huxu, Qingyuan Ma and Guangxi Xiayan chickens affect the IMF content [24]. In contrast with previous studies, three variations of the two breeds examined in this study were detected as follows: G939A, G982A and C1014T. One possible reason for the discrepancy in these results is that the chicken breeds used in our respective experiments have a different genetic background [41]. The IMF content in the chest muscle of HTBC with the TT genotype was increased compared with that of the TC genotype. Thus, the G982A mutable site could be considered as a gene marker for selecting HTBC with increased IMF content in the chest muscle.

H-FABP participates in the transport procedure of fatty acids to the mitochondria during β-oxidation and exists in organs involved in high acid oxidation activity, such as skeletal muscle and cardiac muscle [27]. Our experimental results confirmed that *H-FABP* is expressed in various types of tissues, such as cardiac, breast and thigh muscles [10, 41–44]. Our findings indicate that the *H-FABP* mRNA level in the cardiac muscle of TYCs was increased compared with that in HTBCs. This result is consistent with the results of Wang *et al.*, who reported that the *H-FABP* mRNA expression level in the cardiac muscle of Bai’er layers was increased compared with that in a fat line broiler at the age of 42 d [24].

Our findings demonstrated negative correlations between the *H-FABP* mRNA expression and IMF content in the three tissues of the two chicken breeds with the exception of the leg muscle of TYC. These findings are consistent with those of Tu *et al*., who indicated that the *H-FABP*mRNA expression level has a significant negative effect on the IMF of the cardiac, breast and leg muscles in Rugao and Luyuan chickens [10]. The results are also consistent with the results of Li *et al.,* who reported that high *H-FABP* mRNA expression was correlated with low leg IMF content at 70 d in Baijing You chickens [25]. Furthermore, relatively increased *H-FABP* mRNA expression improved fatty metabolic activity and decomposed fat to produce more energy to satisfy the needs for growth and diversified physiological demands.

The *H-FABP* mRNA and protein expression trends were consistent in the current study, but no significant difference was observed at the protein level. *H-FABP* gene transcription and translation can directly or indirectly affect the synthesis and regulation of proteins in fatty acid metabolism [45]. Tissue-specific expression of *FABP* genes is considered to be primarily regulated at the transcriptional level [45]. Glatz *et al.* reported that *H-FABP* expression was mainly regulated via the process of transcription [46], and this viewpoint was confirmed in pigs [7]. Tyra *et al.* also drew a similar conclusion that the higher expression amount of mRNA was not consistent with higher H-FABP protein levels in pigs [22]. These results indicated that the distribution of fat among different fat deposits might be controlled by different mechanisms and possibly by different genes [47]. This finding indicates a low correlation between *H-FABP* mRNA and protein levels which is in agreement with the relationship between *H-FABP* mRNA level and protein expression level in pigs. [48].

Increasing the IMF content is economically desirable in chicken breeding [40]. Regarding the Chinese indigenous breed HTBC, the most important task would be to protect those genetic resources that have high IMF contents to ensure that precious experimental materials could be used for further study of *H-FABP* or other genes related to IMF content.

## Conclusion

In conclusion, our results suggest that IMF content in the same tissues of HTBC is increased compared with TYC. The G982A mutational site could serve as a genetic marker for increased IMF content in selecting for the chest muscle of HTBC. *H-FABP* gene transcription had a negative impact on IMF content in the two chicken breeds.
